# The Triterpenoid Nrf2 Activator, CDDO-Me, Decreases Neutrophil Senescence in a Murine Model of Joint Damage

**DOI:** 10.3390/ijms24108775

**Published:** 2023-05-15

**Authors:** Kristiana M. Amirova, Petya A. Dimitrova, Milena N. Leseva, Ivanka K. Koycheva, Albena T. Dinkova-Kostova, Milen I. Georgiev

**Affiliations:** 1 Laboratory of Metabolomics, The Stephan Angeloff Institute of Microbiology, Bulgarian Academy of Sciences, 4000 Plovdiv, Bulgaria; kristiana.amirova@gmail.com (K.M.A.); vkoy4eva@abv.bg (I.K.K.); 2 Center of Plant Systems Biology and Biotechnology, 4000 Plovdiv, Bulgaria; 3 Department of Immunology, The Stephan Angeloff Institute of Microbiology, Bulgarian Academy of Sciences, 1113 Sofia, Bulgaria; petya_dimitrova@web.de (P.A.D.); mlesseva@gmail.com (M.N.L.); 4 Division of Cellular and Systems Medicine, School of Medicine, University of Dundee, Dundee DD1 9SY, UK; a.dinkovakostova@dundee.ac.uk; 5 Department of Medicine and Pharmacology and Molecular Sciences, School of Medicine, Johns Hopkins University, Baltimore, MD 21205, USA

**Keywords:** triterpenoids, CDDO-Me, Nrf2, neutrophils, senescence, CXCR4, CD11b

## Abstract

The synthetic 2-cyano-3,12-dioxo-oleana-1,9(11)-dien-28-oic acid methyl ester (CDDO-Me) is a potent activator of the erythroid 2-p45-derived factor 2, Nrf2, a leucine-zipper regulator of the antioxidant response. Herein, we investigated the effect of CDDO-Me on neutrophil function in a murine model of joint damage. Collagenase-induced osteoarthritis (CIOA) was initiated by the intra-articular injection of collagenase in the knee-joint cavity of Balb/c mice. CDDO-Me was administrated intra-articularly twice a week starting at day 7 post-CIOA, and its effect was evaluated at day 14. Neutrophils in blood and bone marrow (BM), cell apoptosis, necrosis, expression of C-X-C chemokine receptor 4 (CXCR4), beta-galactosidase (β-Gal), and Nrf2 levels were measured by flow cytometry. In vitro, CDDO-Me promoted cell survival, reduced cell necrosis, and increased Nrf2 levels by 1.6 times. It decreased surface CXCR4 expression and reduced the frequency of senescent β-Gal+CXCR4+ neutrophils by three times. In vivo, the degree of knee-joint damage in CIOA was correlated with upregulated CXCR4 on CD11b+ neutrophils. CDDO-Me improved the disease histological score, increased the levels of Nrf2, and downregulated surface CXCR4 on mature BM cells. Our data suggest that CDDO-Me may act as a potent regulator of neutrophil senescence during the progression of knee-joint damage.

## 1. Introduction

Cellular senescence is a complex state of cell cycle arrest during which cells remain viable, but acquire a robust senescence-associated secretory phenotype (SASP) that can alter the structure and function of the surrounding cells and tissues. Widely used senescent markers for transcriptionally active cells are elevated cyclin-dependent kinase inhibitor 2A (p16-Ink4a) levels, positive staining for senescence-associated β-galactosidase, and telomere shortening [1,2]. Cellular senescence can be detrimental, associated with functional loss in age-related and degenerative diseases [2,3], and the clearance of senescent cells improves these disease pathologies [2,3].

Osteoarthritis (OA) affects all tissues and cells in the joints [4]. Besides the slow metabolic imbalance in the cartilage and bone, chronic joint inflammation is present during all disease stages. Inflammation begins with the recruitment of neutrophils and macrophages to the synovium and production of cytokines, chemokines, pro-inflammatory mediators, and matrix metalloproteinases (MMPs). The process is sustained by an engagement of toll-like receptor (TLR) 4 by lipopolysaccharide (LPS), originating from the microbiome [5,6] and/or by disease associated molecular patterns (DAMPs), present in the OA synovium during disease progression [7]. An inflammatory environment further reinforces the SASP phenotype and cell cycle arrest program in chondrocytes, synovial fibroblasts, and bone cells [8].

Recently, the role of Nrf2 in the development of osteoarthritis [9], as well as in aging and cellular senescence [10], has been reported. Nrf2 is a transcriptional regulator of genes involved in oxidative stress response, antioxidant defense, cellular detoxification, drug transport, and cytoprotection [11,12,13,14,15]. During the normal cellular state, Nrf2 is unstable, continuously degraded following ubiquitination by the Kelch-like ECH-associated protein 1 (Keap1)/Cullin 3 E3 ubiquitin ligase complex [16]. Under stress, Nrf2 is rapidly accumulated and translocated to the nucleus where it affects the expression of more than 1200 directly targeted genes [15]. 

The semi-synthetic triterpenoids CDDO-Im (imidazolide derivative) and CDDO-Me (methyl ester derivative) are potent activators of Nrf2 and Nrf2-regulated genes [17,18,19]. Triterpenoids contain six five-carbon isoprene units and are synthesized in plants by the cyclization of squalene [20]. CDDO-Me has well known anti-angiogenic, anti-cancer, anti-inflammatory, and hepatoprotective properties [21,22,23]. It has the chemical structure of the bardoxolone methyl ester, as shown in the PubChem Database (Pub Med CID: 400769; Supplementary Figure S1). CDDO-Me inhibits cartilage loss and osteoclastogenesis in a destabilized medial meniscus mouse model of OA, demonstrating its efficiency to attenuate local destruction by bone and cartilage cells [24]. However, the effect of CDDO-Me on immune cells in OA has not been evaluated and data about the role of the compound on immune cell senescence in OA are still limited. 

Neutrophils in OA synovial fluids show specific characteristics such as increased production of reactive oxygen species and phagocytic activity [25]. In our previous study, we demonstrated that neutrophil depletion in an experimental model of OA improves the disease progression [26], suggesting that the therapy approach targeting neutrophil functions might be beneficial for OA. 

Herein, we investigated the role of CDDO-Me on the function of bone-marrow-derived neutrophils in an experimental model of OA. Using flow cytometry, we examined the in vitro compound’s action on Nrf2 cytoplasmic levels, p38 phosphorylation, CXCR4 expression on CD11b+ mature neutrophils, and cell migration toward the CXCR4 ligand. Immunofluorescence was used to assess the co-expression of CXCR4 and β-galactosidase on CDDO-Me treated/untreated CD11b-enriched neutrophils, indicative of the senescence phenotype. In mice with collagenase-induced osteoarthritis (CIOA), we investigated the effect of the intra-articular administration of CDDO-Me on Nrf2 levels and the presence of senescent CD11b^high^CXCR4+ mature neutrophils in bone marrow and blood. Our study aimed to prove that CDDO-Me-induced senescence of bone-marrow-derived neutrophils had therapeutic potential in OA. 

## 2. Results

### 2.1. Effect of CDDO-Me on Caspase-Mediated Apoptosis Measured by Poly [ADP-Ribose] Polymerase 1 (PARP-1) Cleavage and on Cell Necrosis in Late Cultures

**Figure 1:**
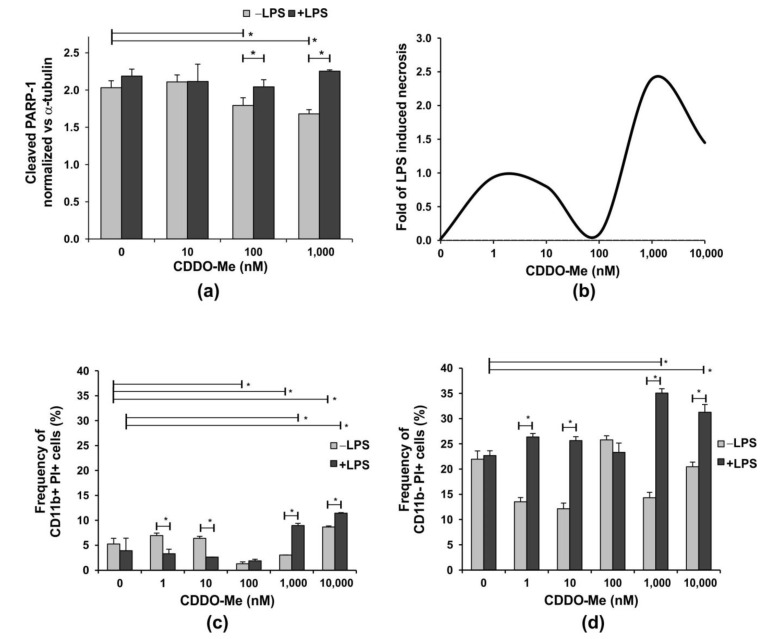
The effect of CDDO-Me on the apoptosis of bone-marrow-derived neutrophils. BM neutrophils were stimulated with 100 ng/mL LPS in the presence of increasing concentrations of CDDO-Me for 18 h. (**a**) PARP-1 cleavage was used as a surrogate marker for caspase activation and apoptosis. The level of cleaved PARP-1 was evaluated by the colorimetric Pierce™ Cleaved PARP In-Cell kit (#62219; Thermo Fisher Scientific, Waltham, MA, USA) and was normalized to the level of α-tubulin according to the manufacturer’s protocol. Data represent the mean ± SD of two independent experiments, involving three mice/experiment; samples run in triplicate (*n* = 6/group), * significant *p* values between the groups, using the ANOVA test. (**b**) Fold of LPS-induced necrosis in one representative experiment evaluated using propidium iodide (PI) staining using flow cytometry (BSR II flow cytometer) with BD FACSDiva v6.1.2 Software (Becton Dickinson GmbH, San Jose, CA, USA). Neutrophils were cultured as described in (**a**) for 24 h. Flow cytometry analyses showing the effect of increasing CDDO-Me concentrations on the frequency of (**c**) CD11b+ PI+ and (**d**) CD11b− PI+ cells cultured as described (**a**) for 24 h. PI staining was performed at the end of culture period and after cell incubation with the APC-labelled antibody against CD11b. Flow cytometry data were obtained using a BSR II flow cytometer with BD FACSDiva v6.1.2 Software (Becton Dickinson GmbH, San Jose, CA, USA) and analyzed using Cyflogic software 1.2.1 (CyFlo Ltd., Turku, Finland). The data represent mean ± SD of two independent experiments, involving three mice/experiment; and samples run in duplicate (*n* = 6/group), * significant *p* values between the groups, using the ANOVA test.

Caspase-mediated apoptotic cell death involves the cleavage of several key proteins required for cellular function and survival [27]. PARP-1 is one of the cellular substrates of caspases and its overactivation and cleavage is a hallmark of apoptosis [28]. Hence, we started by investigating whether or not CDDO-Me induces PARP-1 cleavage and, thus, cell apoptosis. We also studied the apoptotic effect of CDDO-Me on LPS-stimulated/unstimulated neutrophils because a recent study indicated that neutrophil response to LPS is a marker of altered neutrophil function and aging [29]. Purified BM neutrophils from healthy mice were activated with 100 ng/mL LPS in the absence or presence of 10 nM, 100 nM, and 1000 nM CDDO-Me for 18 h. LPS stimulation did not significantly increase the level of cleaved PARP-1 compared with the untreated control cells (Figure 1a). The Nrf2 activator decreased the amount of cleaved PARP-1 in non-stimulated neutrophils in a dose-dependent manner, improving cell survival (Figure 1a). However, this effect of CDDO-Me was masked by LPS stimulation (Figure 1a), which likely triggers downstream anti-apoptotic pathways. A recent paper proposed a model of dead decision of neutrophils where apoptosis is triggered by inflammatory stimuli, but when the insult is strong or persistent, neutrophils undergo necrosis causing tissue damage [30]. We confirmed that prolonged neutrophil culture for 24 h (6 h longer than in the previous experiment, late cultures) in the presence of strong activator, such as LPS, significantly increased necrosis, evaluated by propidium iodide (PI) staining (Figure 1b and Supplementary Figure S2). 

CDDO-Me at a concentration of 100 nM inhibited this process in late cultures, while the compound’s concentrations higher than 1000 nM (1 µM) failed to reverse LPS-induced necrosis (Figure 1b). In our study, bone marrow neutrophils were divided into two subpopulations with distinct CD11b expressions. CD11b+ cells represent pre-neutrophils, namely immature and mature neutrophils that were resistant to necrosis, while CD11b− cells dowregulated the receptor and showed elevated necrosis in the late cultures (Figure 1c,d). The CDDO-Me, 100 nM, reduced the frequency of CD11b+ necrotic cells to less than 2% and failed to affect CD11b− necrotic cells in LPS-stimulated or unstimulated cells (Figure 1c,d). In contrast, at higher concentrations, the compound elevated LPS-induced necrosis not only in CD11b− neutrophils, but also in more resistant CD11b+ cells. At low concentrations, CDDO-Me sustained lower frequencies of CD11b− necrotic cells in non-stimulated cultures (Figure 1d). 

### 2.2. CDDO-Me Increases Nrf2 Levels, and Decreases Surface CXCR4 Expression and p38 Mitogen-Activated Protein Kinase (MAPK) Phosphorylation in BM-Derived Neutrophils

**Figure 2:**
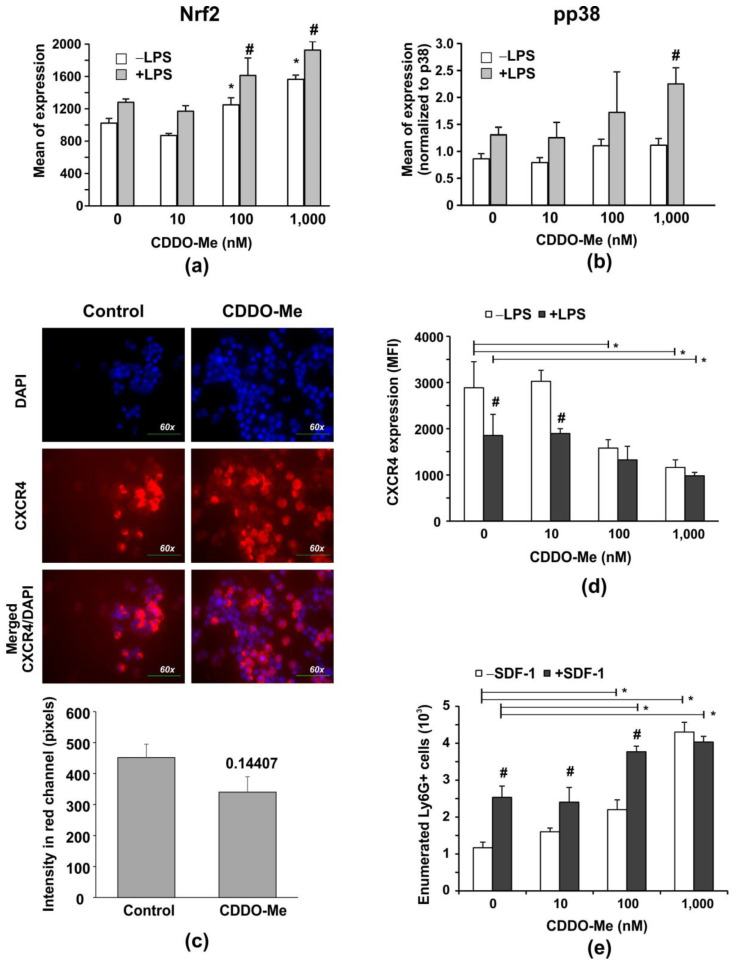
CDDO-Me increases Nrf2 levels in LPS-stimulated BM-derived Ly6G+ neutrophils, and down regulates CXCR4 expression affecting chemotaxis to SDF-1 and LPS-mediated phosphorylation of p38 MAPK. Purified BM neutrophils were stimulated with 100 ng/mL LPS (#2637, strain O5:B5, Sigma-Aldrich, Munich, Germany) in the presence of increasing concentrations of CDDO-Me for 6 h. The protein levels of Nrf2 (**a**) and of phosphorylated/non-phosphorylated p38 MAPK (**b**), respectively, were measured by flow cytometry using PE-labelled antibody against pT180/pY182, pp38 (clone 4NIT4KK, #612565, eBiosciences, Hatfield, UK), purified antibody against total protein p38 (clone Poly6224; #622403, BioLegend, London, UK), and FITC-labelled anti-rabbit antibody (1:500 diluted; #406403; BioLegend, London, UK). The data were obtained by BD FACSDiva v6.1.2 Software (Becton Dickinson GmbH, San Jose, CA, USA) and analyzed by Flowing Software 2.2 (Cell Imaging Core, Turku Centre for Bio-technology, Turku, Finland). The mean value of phosphorylated p38 MAPK (pp38) protein (*n* = 6/group) was normalized to the mean value of the total p38 MAPK protein (*n* = 6/group). The data represent the mean ± SD of two experiments run in triplicate (*n* = 6/group), # significant *p* values between LPS-stimulated groups (+LPS), * significant *p* values between unstimulated (−LPS) groups, using the ANOVA test. (**c**) Immunofluorescence showing CXCR4 expression on BM neutrophils after 6-h culture in the absence of presence of 1000 nM CDDO-Me. Fluorescence Leica DM6 microscope with DFC7000 T camera and the LAS × 5.0.2 software (Leica Microsystems; RSR Ltd., Sofia, Bulgaria) were used at magnification 60× to capture images from 5 different fields of each sample. Image analysis was performed by Image J software (National Institutes of Health, University of Wisconsin) and expressed as fluorescence intensity in the red channel. Graph shows mean ± SD of the data (*n* = 15/group), * number denotes *p* value between the groups, using Student *t*-test. (**d**,**e**) Purified BM neutrophils were activated with 100 ng/mL LPS in the presence of increasing concentrations of CDDO-Me for 6 h. Surface CXCR4 expression shown as mean fluorescence intensity (MFI) was assessed by flow cytometry using APC-labelled CXCR4 (clone L276F12, #146507, BioLegend, London, UK). (**d**). Data represent a mean ± SD of four experiments, including three mice, samples run in triplicate (*n* = 12/group). # significant *p* values between unstimulated (−LPS) and LPS-stimulated cells (+LPS), * significant *p* values between CDDO-Me-treated and untreated groups, using the ANOVA test. (**e**) Effect of CDDO-Me on cell chemotaxis towards SDF-1. BM-derived neutrophils (2 × 10^6^ cells/mL) were pre-treated with increasing concentrations of CDDO-Me for 1 h. The cells were washed, added to an insert, and allowed to migrate for 6 h towards a medium containing 10 ng/mL murine recombinant SDF-1 (#250-20B; PeproTech, Cambridge, UK). The migrated cells were enumerated by counting beads using BD FACSDiva v6.1.2 Software (Becton Dickinson GmbH, San Jose, CA, USA). The data represent the mean ± SD of two experiments, including three mice/experiment, samples (*n* = 6/group) run in triplicate. # significant *p* values between −SDF-1 and +SDF-1 groups, using the ANOVA test. * significant *p* values between CDDO-Me-treated and untreated groups, using the ANOVA test.

In order to confirm that CDDO-Me acts as an Nrf2 activator in our experimental conditions, we stimulated purified BM-derived neutrophils with 100 ng/mL LPS in the absence or presence of increasing concentrations of CDDO-Me (10, 100, and 1000 nM) for 6 h. We observed that CDDO-Me elevated the protein level of Nrf2 in both unstimulated and LPS-stimulated cells in a dose-dependent manner (Figure 2a). In neutrophils treated with LPS but not in unstimulated cells, CDDO-Me increased p38 MAPK phosphorylation at the highest concentration (Figure 2b), indicating that LPS-dependent pathways were activated along with Nrf2 activation. However, the p38 MAPK inhibitor SB203580 failed to lower cytoplasmic Nrf2 in LPS-stimulated cells cultivated in the presence of 1000 nM CDDO-Me, implying that p38 MAPK phosphorylation was indirectly affected by the activator (see Supplementary Figure S4). 

Several lines of evidence exist about the role of Nrf2 in aging [31]. In neutrophils, the process of aging is associated with the acquisition of the senescence phenotype, which is characterized by the upregulation of the chemokine receptor CXCR4 [32]. The effect of CDDO-Me on CXCR4 expression was assessed by immunofluorescence and flow cytometry (Figure 2c–e). The compound-treated group showed decreased CXCR4 staining in contrast with the vehicle-treated group (Figure 2c). To confirm the obtained data, we evaluated the surface CXCR4 expression using flow cytometry. We found that LPS stimulation (100 ng/mL for 6 h) upregulated CXCR4 expression (Figure 2d). CDDO-Me induced a dose-dependent CXCR4 downregulation in both LPS-stimulated and in unstimulated cells (Figure 2d). One study demonstrated that CXCR4 deficiency decreases LPS-induced p38 MAPK phosphorylation [33], which contradicts our findings. We observed that 1000 nM CDDO-Me decreased LPS-induced CXCR4 expression but enhanced LPS-induced p38 MAPK phosphorylation (Figure 2b, see Supplementary Figure S5). However, the effect of 1000 nM CDDO-Me on LPS-induced CXCR4 downregulation was unrelated to p38 MAPK activation, as the p38 MAPK inhibitor SB203580 was unable to restore CXCR4 expression on CDDO-Me and LPS-treated cells in contrast with the control cultures (see Supplementary Figure S5). 

**Figure 3:**
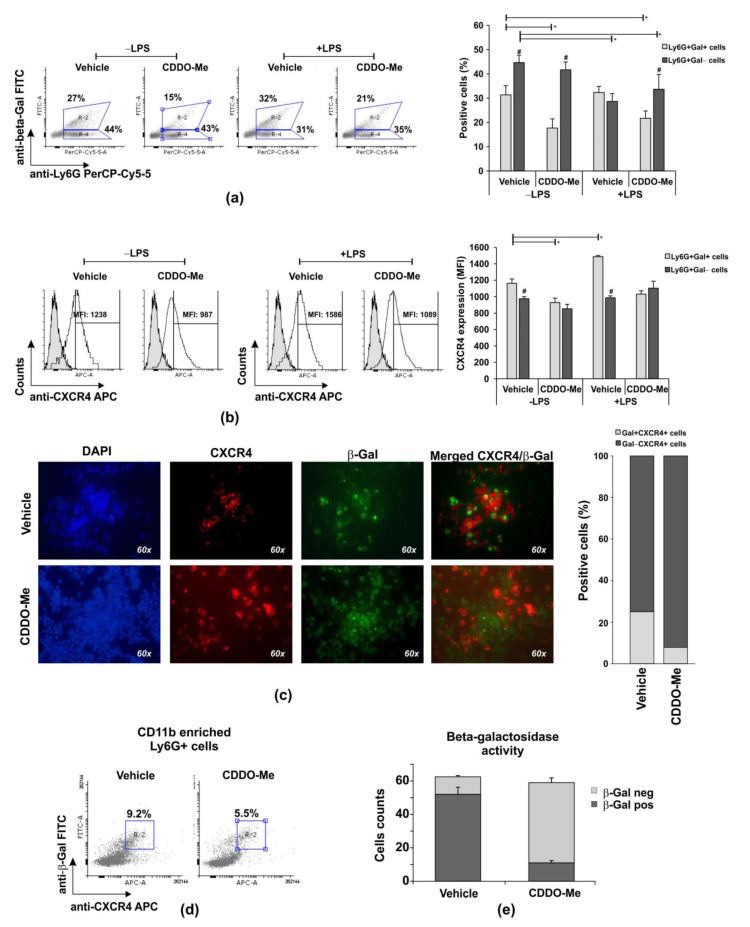
Effect of CDDO-Me on senescence of Ly6G+CD11b+ cells. (**a**) Flow cytometry analyses of CD11b+-enriched BM neutrophils positive for β-Gal in a representative experiment. The data were obtained using BD FACSDiva v6.1.2 Software (Becton Dickinson GmbH, San Jose, CA, USA) and analyzed using Flowing Software 2.2 (Cell Imaging Core, Turku Centre for Biotechnology, Turku, Finland). The graph shows the mean ± SD from the experiments, including three mice, samples (*n* = 6/group) run in triplicate; * significant *p* values between β-Gal+ groups; # significant *p* values between β-Gal groups, using an ANOVA test. (**b**) CXCR4 expression on Ly6G+Gal+ cells in a representative histogram panel. The graph shows the mean ± SD from two experiments, including three mice, samples (*n* = 6/group) run in triplicate. * significant *p* values between β-Gal groups, using the ANOVA test. (**c**) CXCR4 and β-Gal-positive cells evaluated using a fluorescence Leica DM6 microscope (DFC7000 T camera; LAS × 5.0.2 software) at magnification 60× (Leica Microsystems; RSR Ltd., Sofia, Bulgaria). Images were taken from five different fields of each sample using appropriate filters and exposure, and were then merged (*n* = 6/group). Image analysis was performed using Image J software (National Institutes of Health, University of Wisconsin) after defining the threshold of each signal—red, blue, and green. The graph represents the proportion of double positive cells within a total of 60 counts/field (*n* = 6/group). (**d**) Representative histograms out of six per group demonstrating the effect of 1000 nM CDDO-Me on frequencies of Ly6G+CD11+CXCR4+Gal+ neutrophils in a 6 h culture evaluated using flow cytometry. The data were obtained by BD FACSDiva v6.1.2 Software (Becton Dickinson GmbH, San Jose, CA, USA) and analyzed by Cyflogic software 1.2.1 (CyFlo Ltd., Turku, Finland). (**e**) Effect of 1000 nM CDDO-Me on the counts of CD11b+ neutrophils positive for β-galactosidase activity. Neutrophils were cultured with the chromogenic buffer, then fixed and air-dried. The positive cells for β-galactosidase were examined by Leica DMi8 inverted microscope at 10× and 20× magnifications using Flexacam C1 camera (Leica Microsystems; RSR Ltd., Sofia, Bulgaria). The images were captured and the cells showing β-galactosidase activity (blue staining) were detected and counted using Image J software (National Institutes of Health, University of Wisconsin). Data represent mean ± SD from two experiments, samples run in triplicate (*n* = 6/group). Five images were captured from each sample at different fields and wells (total samples 30/group), using Student’s *t*-test.

An in vitro chemotaxis assay was carried out to determine whether CDDO-Me modified the BM neutrophils’ responsiveness and chemotaxis toward the receptor ligand stromal cell-derived factor 1 (SDF-1) (Figure 3d). We observed that BM-derived neutrophils migrated towards SDF-1 within 6 h of incubation (Figure 3e). CDDO-Me at high concentrations (100 and 1000 nM) stimulated Ly6G+ cell migration towards SDF-1 (Figure 2d).

### 2.3. CDDO-Me Effects Neutrophil Senescence

We observed that Ly6G+CD11b+ cells were more resistant to necrosis than Ly6G+CD11b− cells in late cultures, and CDDO-Me at concentrations between 100 and 1000 nM significantly affected the survival of the CD11b+ population (Figure 1). We next purified BM-derived Ly6G+ cells and enriched the population to ~80% CD11b+ cells via magnetic sorting (Supplemented Figure S3a). Neutrophils were then stimulated or not with LPS (100 ng/mL) in the presence of the vehicle (DMSO) or 1000 nM CDDO-Me. In order to identify the senescence phenotype, the cells were collected after 6 h and the intracellular level of β-galactosidase (β-Gal) (Figure 3a–d), β-Gal enzyme activity (Figure 3e), and the surface CXCR4 were assessed (Figure 3b–d). The percentage of unstimulated Ly6G+Gal+ neutrophils (CD11b enriched) decreased by two times in CDDO-Me-treated in comparison with the vehicle-treated cultures (Figure 3a). The frequency of Ly6G+Gal+ cells was modestly enhanced by LPS stimulation (Figure 3a) and maintained with CDDO-Me (1000 nM; Figure 3a). In fact, when we studied the density of the surface CXCR4, defined as an indicator of neutrophil senescence, we noticed that vehicle-treated Ly6G+Gal+ cells had a higher chemokine receptor expression than CDDO-Me-treated Ly6G+Gal+ cells (Figure 3b). We then conducted immunofluorescence analyses to identify CXCR4+ cells that express β-galactosidase. Vehicle-treated Ly6G+CD11b+ cells showed co-staining for CXCR4 and β-Gal more frequently than CDDO-Me-treated cells. The numbers of Gal+CXCR4+ cells were three times higher in the vehicle group than in the CDDO-Me-treated group (Figure 3c). According to additional flow cytometry data, CDDO-Me reduced senescent Ly6G+CD11b+CXCR4+Gal+ frequency by around 1.8 times (Figure 3e). We next studied the activity of β-Gal in a 6 h culture of CDDO-Me treated or untreated CD11b-enriched Ly6G+ cells (Figure 3e). Neutrophils positive for the enzyme activity were identified by the color response in blue after the addition of a substrate solution (Supplemented Figure S3b). Blue cells were counted in the captured photographs using an Image J software plug in. Beta-Gal activity was detected in 83% of cells cultured in the presence of the vehicle and in ~17% of neutrophils cultured in the presence of CDDO-Me (Figure 3e). Altogether, the data showed that CDDO-Me inhibited the senescence phenotype of Ly6G+CD11b+ neutrophils. 

### 2.4. CDDO-Me Inhibits Disease Activity and MMP-9 and TNF-α Production in Synovial Tissue Extracts

**Figure 4:**
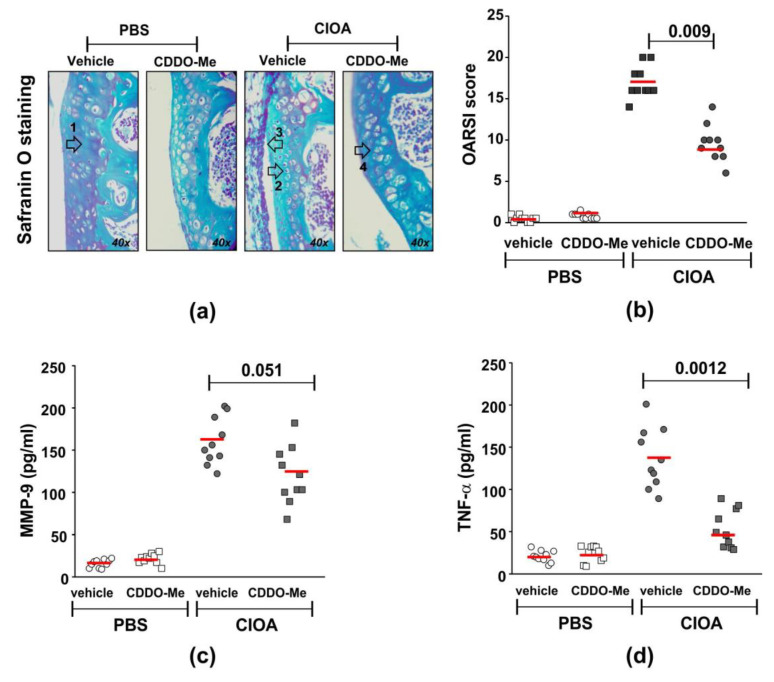
Effect of CDDO-Me on disease activity and MMP-9 and TNF-α production in synovial tissue extracts. CIOA was induced in Balb/c mice by injection in the knee cavity of 5 U/10 µL of collagenase from *Clostridium histolyticum* (CIOA group) or 10 µL of endotoxin-free PBS (PBS group). (**a**) Representative photographs of Safranin O-stained knee-joint sections from CDDO-Me and vehicle-treated CIOA mice showing tibial cartilage with chondrocytes. Arrows point to the following: (1) proteoglycan, (2) chondrocyte apoptosis, (3) inflammation, and (4) chondrocyte proliferation. Images were captured using a light microscope (Boeco BM-800, Hamburg, Germany) at 40× magnification by a Nikon camera (Nikon Inc, Melville, NY, USA). (**b**) Effect of CDDO-Me on histological OARSI score calculated upon blinded evaluation using a light microscope (Boeco BM-800, Hamburg, Germany) at 20× and 40× magnifications of Safranin O-stained knee-joint sections harvested at 60 μm interval, from total 10 sections/mouse (*n* = 10 mouse/group). The red line represents the mean value in the group. The numbers on the graphs denote the *p* value between CDDO-Me-treated and untreated groups (*n* = 10/group), using ANOVA test. (**c**) Effect of CDDO-Me on MMP-9 production in synovial tissue harvested at day 14 of CIOA. The secreted MMP-9 was evaluated the indirect ELISA method using the Quantikine ELISA kit of R&D System (#MMPT90; R&D System, Abingdon, UK)). Data represent individual values from *n* = 10 mice/group; samples run in triplicate in the assay. The red line represents the mean value in the group. The numbers on the graphs denote the *p* value between CDDO-Me-treated and untreated groups, using Student’s *t*-test. (**d**) Effect of CDDO-Me on TNF-α production in synovial tissue extracts harvested at day 14 of CIOA. The secreted TNF-α was evaluated using indirect ELISA method using the ELISA MAX™ Deluxe kit (#430904; BioLegend, London, UK). Data represent individual values from *n* = 10 mice/group, samples run in triplicate in the assay. The numbers on the graphs denote *p* values between CDDO-Me-treated and untreated groups, using Student’s *t*-test.

We next studied the in vivo effect of CDDO-Me in mice with CIOA. The compound was injected intra-articularly in anesthetized Balb/c mice, twice a week starting at day 7 post-CIOA (50 µg/knee). At day 14, knee joints were collected and used for histology or synovial tissue extract preparation. We observed a significantly decreased OARSI score for knee-joint damage in the CDDO-Me group in comparison with the vehicle-treated CIOA mice (Figure 4a,b). The disease improvement was accompanied by the inhibition of locally produced degradation enzyme MMP-9 (Figure 4c) and especially the pro-inflammatory cytokine TNF-α (Figure 4d).

### 2.5. Correlation between Disease Activity in CIOA Mice and CXCR4 Expression on BM-Derived Ly6G+ Neutrophils

**Figure 5:**
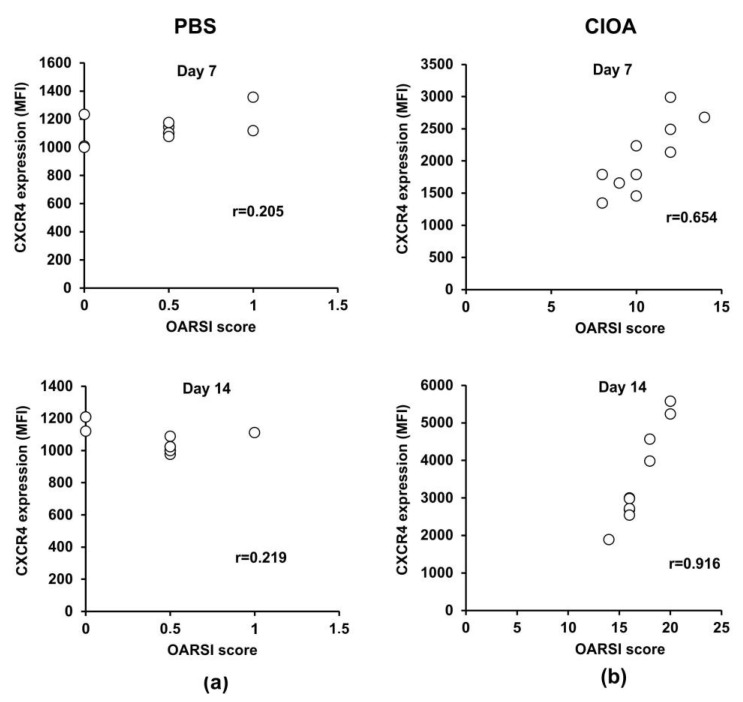
Correlation between disease activity and CXCR4 expression on BM-derived Ly6G+ neutrophil cells. (**a**) Pearson’s correlation between OARSI score and CXCR4 expression in PBS control group at day 7 (*n* = 10/group from two separate experiments) and day 14 (*n* = 10/group from two separate experiments). (**b**) Pearson’s correlation between disease activity and CXCR4 expression in the CIOA group at day 7 and day 14. The coefficient was calculated using plug-in Real Statistic and the null hypothesis was accepted or rejected when α = 0.05 at degree of freedom 16.

Recently, we reported that neutrophil depletion in mice with collagenase-induced osteoarthritis attenuated disease activity [26]. Herein, we exploited the same experimental model to study whether or not CXCR4 expression on BM-derived neutrophils is associated with the degree of knee-joint injury. CIOA was induced by injecting both knees of Balb/c mice with collagenase (5 U/knee). At day 7 and day 14 we collected right knee joints for histology and the left tibia for the isolation and purification of BM neutrophils. We performed OARSI histological analysis to evaluate the degree of joint damage, while also measuring the expression of CXCR4 on neutrophils that highly express CD11b, a marker for terminally differentiated and mature cells (Figure 5a,b). At day 7 and day 14, Pearson’s correlation coefficients calculated for the CIOA group showed a strong positive correlation (degree of freedom 16, α = 0.05) between both parameters, disease activity and CXCR4 expression on neutrophils (Figure 5b). The data indicated that the progression of the disease was associated with the acquisition of the senescence CXCR4+ phenotype by CD11b^high^ neutrophils in the bone marrow.

### 2.6. Effect of CDDO-Me on Blood-and BM-Neutrophil Pools in CIOA Mice and on Nrf2 Intracellular Level in CIOA Neutrophils

**Figure 6:**
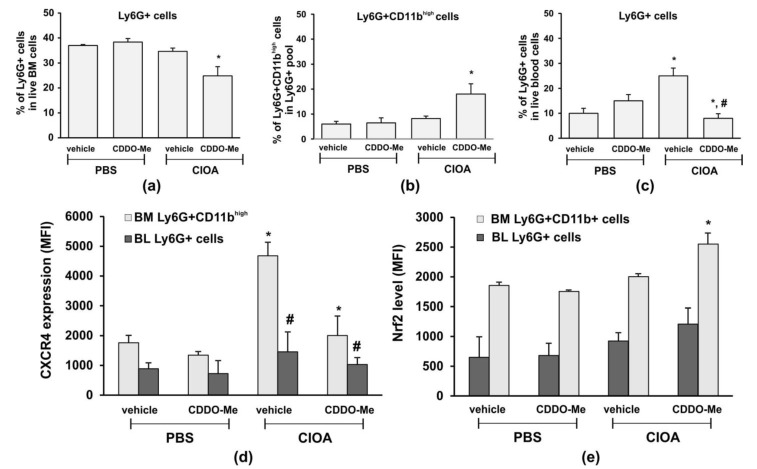
CDDO-Me changes the frequency of Ly6G+ cells in the circulation and in the BM and down regulates CXCR4 expression. (**a**) Total frequency of Ly6G+ cells in the marginal live BM pool following treatment with CDDO-Me of CIOA mice. The data were obtained using BD FACSDiva v6.1.2 Software (Becton Dickinson GmbH, San Jose, CA, USA) and analyzed by Flowing Software 2.2 (Cell Imaging Core, Turku Centre for Bio-technology, Turku, Finland). Data represent a mean ± SD of two experiments with *n* = 7 mice/group. Each sample from one mouse was run in triplicate, * significant *p* values between CDDO-Me-treated and non-treated CIOA mice, using Student’s *t*-test. (**b**) Effect of CDDO-Me on the frequency of mature neutrophils Ly6G+/CD11b^high^, evaluated by flow cytometry as described in (**a**). Data represent a mean ± SD of two experiments with *n* = 5 mice/group. Each sample from one mouse was run in triplicate, * significant p values between CDDO-Me-treated to non-treated CIOA groups, using Student’s *t*-test. (**c**) Effect of CDDO-Me on blood neutrophils in CIOA mice, evaluated by flow cytometry as described in (**a**). Data represent mean ± SD of two experiments with *n* = 7 mice/group. Each sample from one mouse was run in triplicate, * significant p values between CIOA and PBS groups, # significant p values between CDDO-Me-treated and non-treated CIOA groups, using Student’s *t*-test. (**d**) Effect of CDDO-Me on CXCR4 expression on mature BM-derived neutrophils and circulatory blood Ly6G+ cells evaluated by flow cytometry as described in (**a**). Data present a mean ± SD of two experiments with *n* = 7 mice/group. Each sample from one mouse was run in triplicate, * significant p values between CIOA and PBS groups, # significant *p* values between CDDO-Me-treated and non-treated CIOA groups, using Student’s *t*-test. (**e**) CDDO-Me increases the Nrf2 level in mature BM neutrophils. The Nrf2 levels in blood (BL) and bone marrow (BM)-derived neutrophils are measured by flow cytometry, as described in (**a**). Data present mean ± SD of two experiments with *n* = 7 mice/group. Each sample from one mouse was run in triplicate, * significant *p* values between CIOA and PBS groups, using Student’s *t*-test.

As we observed that disease activity in CIOA mice was correlated with the senescent phenotype in CD11b^high^ BM-derived neutrophils (Figure 5), we next delineated the effect of CDDO-Me on the number and frequency of Ly6G+ cells in blood- and BM-derived neutrophils from CIOA mice. Treatment with CDDO-Me decreased the frequency of Ly6G+ cells in BM (Figure 6a). However, we found an elevated frequency of mature neutrophils (Ly6G+/CD11b^high^) within the BM pool (Figure 6b). In blood, CDDO-Me significantly reduced the number of circulating neutrophils (Figure 6c) and inhibited the senescent neutrophil phenotype as assessed by the CXCR4 expression (Figure 6d). We hypothesized that CDDO-Me may stimulate the homing of senescent neutrophils from the blood to the bone marrow. We next investigated whether or not the local application of CDDO-Me affected the Nrf2 level in mature neutrophils from the bone marrow (Figure 6e). Neutrophils were isolated from the CIOA tibia, which was affected by the localized pathological process (Figure 4a—chondrocyte apoptosis). We observed that CDDO-Me was able to significantly increase the Nrf2 levels in mature BM-derived neutrophils from CIOA mice. Blood neutrophils were less responsive to the effect of CDDO-Me and showed only slightly elevated Nrf2 (Figure 6e). 

## 3. Discussion

Neutrophils are short-lived cells prone to acquire the senescence phenotype faster than other immune cells. Senescent neutrophils express CXCR4 and return to bone-marrow niches enriched with stromal SDF-1. The cells undergo constitutive apoptosis and are eliminated by bone marrow macrophages, which forward signals to stimulate granulopoiesis and cell egress from the bone marrow to circulation [34]. The homeostatic regulation of neutrophil generation, mobilization from bone marrow, migration in periphery, and spontaneous apoptosis is a key multistep mechanism to control protective and detrimental immune responses. We previously showed that the depletion of neutrophils in CIOA attenuated cartilage damage preventing disease progression [26]. The role of the cells at different stages of OA development has also been described by other groups [4,25,34]. Neutrophils can contribute to OA pathology by (i) participating in local chronic inflammation via secreted cytokines, pro-inflammatory mediators, and MMPs, all promoting cartilage damage and chondrocyte apoptosis and likely sustaining the SASP phenotype in all cells in the OA synovium, and/or (ii) altering their spontaneous apoptosis leading to compensatory changes in granulopoiesis, subsequent mobilization from BM and intensive migration to OA synovial fluid maintaining inflammation at late stages, and/or (iii) altering their senescence, which may impose additional functional changes in the neutrophil phenotype in synovial fluid and BM. Neutrophil senescence in BM can indirectly affect granulopoiesis, which may change the structure and functionality of bone marrow niche, which is a source of osteoclast precursors. So far, there are no data showing a role of neutrophils senescence in OA pathology, nor direct evidence for altered bone marrow precursor niches in OA by senescent neutrophils in BM. 

Herein, we found that the Nrf2 activator CDDO-Me increased neutrophil cell survival via the suppression of caspase-dependent PARP-1 cleavage. This effect of the activator was observed in unstimulated cells and was masked by stimulation with LPS, which likely triggers downstream anti-apoptotic pathways. PARP-1 is a substrate for several “suicidal” proteases such as caspases and MMPs that cleave the protein to various fragments with different molecular weights [27,28]. PARP-1 is cleaved not only during apoptosis, but also during necrosis [35], and can be involved in other neutrophil functions, including chemotaxis, as observed in PAPR-1-deficient neutrophils [36]. Neutrophils can make dead decisions, based on the intensity and strength of inflammatory stimuli [30]. When the signal is strong or persistent as in OA, they undergo necrosis and may contribute to tissue damage [30]. We confirmed that prolonged neutrophil culture for 24 h in the presence of the strong activator, LPS, significantly increased necrosis, determined by PI staining. In late cultures, we also found a population of neutrophils that downregulated CD11b and CD11b− cells, which are sensitive to CDDO-Me action and to necrosis. The downregulation of CD11b may activate the necroptosis pathway involving p38 MAPK and receptor interacting protein kinase-3 (RIPK3) or mixed lineage kinase domain-like protein (MLKL) [37]. Even at low concentrations, CDDO-Me sustained lower frequencies of CD11b− necrotic cells, suggesting that the Nrf2 activator may affect this type of regulated necrosis. Necroptosis of neutrophils may occur in OA, as it can be initiated by death, TLRs and adhesion receptors, exposure to monosodium urate crystals, or phagocytosis of *Staphylococcus aureus* (*S. aureus*), all involved in sterile inflammation and danger signaling [37]. The process can be accelerated in the elderly, where neutrophils spontaneously lose surface CD11b during aging [38]. Hypothetically, CDDO-Me can inhibit sterile inflammation by maintaining low levels of necroptotic CD11b− neutrophils. CD11b+ neutrophils are more resistant to necrosis and 1000 nM CDDO-Me improves their survival and limits their necrosis, suggesting that Nrf2 activation might play a specific role in various types of dead and/or survival neutrophils. 

Triterpenoids are synthesized in plants by the cyclization of squalene, a chain triterpene hydrocarbon and precursor of all steroids [20]. They are widely present in various plants, fruits, and medicinal herbs, including apples, cranberries, figs, olives, oregano, rosemary, and thyme [18,19,20,21]. Many attempts have been made to obtain semi-synthetic oleanane triterpenoids with improved bioavailability and with an anti-cancer activity [22,39,40]. At high concentrations, triterpenoids inhibit the growth and proliferation of various tumors and shorten the survival of cancer cells resistant to conventional chemotherapeutic agents [23,41,42]. At low concentrations, triterpenoids show cytoprotective and anti-inflammatory activities [43]. Two synthetic triterpenoid analogues, CDDO-Im and CDDO-Me, have been described to strongly upregulate the expression of genes, which are part of the Nrf2 regulatory network involved in ROS detoxification, glutathione synthesis, and NADPH regeneration [18,19]. These triterpenoids inhibit LPS-induced oxidative stress and inflammatory cytokine response (interleukin-6 and tumor necrosis factor (TNF)-α production) in murine and human neutrophils [41,42]. It has been shown that CDDO-Im and CDDO-Me can upregulate Nrf2-dependent antioxidant genes such as heme oxygenase 1 (HO-1), glutathione S-transferase, thioredoxin reductase 1, and quinone oxidoreductase 1 in human peripheral blood cells [39,40,41,42,43]. However, CDDO-Im does not affect Nrf2 mRNA levels in the cells, in contrast with CDDO-Me [39,40,41,42,43]. In our study, we used CDDO-Me, which is a potent inducer of cytoprotective genes in the mouse liver, lung, and small intestinal mucosa, and is currently being examined in several clinical trials, including in patients with chronic kidney disease and pulmonary arterial hypertension [44]. The closely related triterpenoid RTA-408 (omaveloxolone) was recently approved by the United States FDA for clinical use in patients with Friedreich’s ataxia [45]. Similar to other cyanoenone triterpenoids, CDDO-Me can affect the Keap1-Nrf2/antioxidant response elements (ARE), nuclear factor kappa B (NF-κB), and signal transducer and activator of transcription 3 (STAT3) signaling [21]. We found that CDDO-Me elevated the levels of Nrf2 in neutrophils and downregulated CXCR4 expression in vivo and in vitro. Thus, it is likely that increased Nrf2 activation by the drug reduced the expression of the senescence marker CXCR4. How is the Nrf2 and CXCR4 cross-talk mediated? In cancer, the co-expression of CXCR4 and Nrf2 has been related to metastasis and a poor prognosis [46], suggesting that an aberrant Nrf2 and CXCR4 cross-talk can be detrimental. Nrf2 binds to ARE elements in the regulatory regions of various genes [13], which can be indirectly or directly involved in CXCR4 signaling and expression in neutrophils. More specifically, oxidant and antioxidant networks are important for the SDF-1/CXCR4 axis and, thus, Nrf2 may indirectly control chemokine receptor expression and internalization via control over the oxidative stress genes. In mesenchymal stem cells, knockout of CXCR4 increases ROS production and activates p38 MAPK and DNA-double strand breaks, thereby decreasing the cellular repopulating potential [47]. Nrf2 activation positively regulates the CXCR4-dependent homing of mesenchymal stem cells [48], but it decreases CXCR4 expression on neutrophils in our study. This difference indicates that various cell types selectively use Nrf2 when carrying out their specific functions. In cells with active transcription, Nrf transcription factors can cross-talk directly with CXCR4-dependent gene promotors and enhancers has been demonstrated for Nrf1 and CXCR4-dependent promoters in the rat retina [49] or in mesenchymal stem cells [48]. However, neutrophils are short-lived cells with restricted de novo transcriptional activity, and CDDO-Me may affect CXCR4 expression in them by interacting directly with other intracellular proteins, including Notch, IkB kinase, NFκB, Akt, transforming growth factor beta (TGF-β), and STAT3, in turn controlling receptor expression/internalization [49]. 

In our study, CDDO-Me inhibited CXCR4 expression and increased the number of Ly6G+ cells migrating towards SDF-1. In the chemotaxis assay, however, neutrophils were pre-treated with CDDO-Me and then exposed to SDF-1. The activator was washed out from the culture, so the CDDO-Me-treated cells migrated more intensively towards the ligand than the non-treated cells. We suggest that CDDO-Me may regulate neutrophil chemotaxis by triggering a compensatory mechanism for chemokine receptor re-expression and/or chemokine production. One potential regulator of neutrophil migration involves the antioxidant enzyme heme oxygenase 1 (HO-1), which can be upregulated by CDDO-Me [21]. It has been shown that HO-1 increases adenosine receptor signaling, which in turn regulates chemokine release and microvascular permeability of neutrophils, and indeed protects tissue from inflammation-related damage [50]. Regarding chemotaxis, SDF-1 itself can induce HO-1 [51] and can activate adenosine receptor signaling promoting spontaneous cell homing and recruitment independently of CXCR4 expression [52]. Finally, a compensatory increase in CXCR4 expression after removal of the Nrf2 activator might be related to a loss of control over CXCR4 signaling, alteration of CXCR4 mRNA stability, or modulation through specific micro RNAs [13]. In vivo, the effect of Nrf2 on the migration of neutrophils may be affected by additional mechanisms involving high mobility group box 1 protein (HBMG1), which may regulate receptor (TLR4 and/or receptor for advanced glycation end product receptor RAGE)-dependent trafficking of inflammatory cells in OA synovial fluid [53]. Nrf2/HBMG1 axes can affect CXCR4 signaling, as well as the migration and proliferation of chondrocyte progenitors, which might be critical in the late stage of OA [54] and should not be excluded as potential mechanism of action of CDDO-Me in CIOA.

HO-1 expression and Nrf2 activation in myeloid cells is dependent on p38 MAPK signaling [47,48]. We observed that CDDO-Me increased p38 MAPK phosphorylation at a higher concentration, as well as Nrf2 cytoplasmic levels, upon LPS stimulation, implying that LPS-dependent pathways were activated alongside Nrf2 activation. One study demonstrated that CXCR4 deficiency decreases LPS-induced p38 MAPK phosphorylation [33], which contradicts our findings. We observed that 1000 nM CDDO-Me decreased LPS-induced CXCR4 expression, but enhanced LPS-induced p38 MAPK phosphorylation. However, the same authors showed that an enhanced p38 MAPK activity is usually sufficient to provoke the SASP phenotype when it is at a high level and when the NF-kB pathway is vigorously activated [33]. Similarly, we observed increased p38 MAPK phosphorylation following strong stimuli via LPS and intensive stimuli via 1000 nM CDDO-Me. It is also important to note that p38 MAPK can play various roles depending on each SASP marker. For example, p38 MAPK is less important for the SASP phenotype triggered by MMPs, but is more involved in the SASP phenotype related to cytokines and chemokines [55,56]. We found that the effect of 1000 nM CDDO-Me on LPS-induced CXCR4 downregulation was unrelated to p38 MAPK activation, as the p38 MAPK inhibitor SB203580 was unable to restore CXCR4 expression on CDDO-Me and LPS-treated cells in contrast with the control cultures. The p38 MAPK inhibitor SB203580 failed also to decrease cytoplasmic Nrf2 in LPS-stimulated cells cultivated in the presence of 1000 nM CDDO-Me, implying that p38 MAPK phosphorylation was indirectly affected by the activator. 

Cellular senescence is a complex process of growth arrest that is not accompanied by cell death [1]. It is an example of evolutionary antagonistic pleiotropy [1]. Senescence has been associated with dysfunctional telomeres, DNA damage, changes in tumor suppressor pathways controlled by the p53 and retinoblastoma proteins, and alterations in chromatin organization [1]. The SASP phenotype is usually described in proliferating cells. In contrast, neutrophils are short-lived differentiated cells with limited transcriptional activity and proliferation, and with little capacity for DNA repair. Neutrophil senescence is observed during aging and is a part of the regulatory circuit of neutrophil pools in the blood, the bone marrow, and in other organs. In vitro, aged mature neutrophils show altered migration, degranulation, and inflammatory capacities through changes in miRNA expression, which controls chemokine and cytokine signaling, small GTPase activity, and the actin cytoskeleton [57,58]. In vitro, we identified the senescence phenotype, by evaluating the intracellular level of β-Gal, β-Gal enzyme activity, and the co-localization of the surface CXCR4 with β-Gal. CDDO-Me decreased the percentage of unstimulated Ly6G+Gal+ neutrophils and maintained the frequency of this population in LPS stimulated cultures. It reduced CXCR4 expression on Ly6G+Gal+ cells and it decreased CXCR4+Gal+ frequency by around 1.8 times. The activator in vitro treatment contributed to reduced counts of cells co-expressing CXCR4 and β-Gal, and decreased Beta-Gal activity by ~17%. Our data show that CDDO-Me attenuated the senescence phenotype and decreased the number of senescent cells. Cellular senescence related to aging may impact the development of chronic inflammatory diseases. We found that the degree of knee-joint damage was correlated with an increased CXCR4 expression in blood and BM-derived neutrophils, probably due to the acquisition of the senescent phenotype of neutrophils. The treatment of mice with the Nrf2 activator CDDO-Me reduced the histological score of joint damage and inhibited the amount of pro-inflammatory TNF-α and the degrading enzyme, MMP-9, in synovial extracts. 

The Nrf2 pathway is actively involved in danger signal machinery through the regulation of the secretion of high mobility group box 1 protein (HMGB1) [59]. In chronic inflammation and OA, danger signals delivered by RAGE promote and maintain cellular senescence [60] and elevated expression of a RAGE ligand, HMGB1, in OA tissues as well as in the nucleus and cytoplasm of late-stage OA chondrocytes [60]. As a result, HMGB1 can contribute to pyroptosis in macrophages and synovial fibroblasts [61,62] by accelerating the cleavage of inactive forms of IL-1β, IL-18, and gasdermin D [63]. Despite the fact that we did not assess HMGB1 levels in synovial fluid or OA tissue, the improvement in CIOA in our experimental model might be associated with reduced danger signal machinery, involving HMGB1 secretion. The research on Nrf2 and HMGB1 has shown that pharmacological Nrf2 activation induces HMGB1 release, which is inflammasome-dependent and occurs as a result of RAGE overexpression [64]. Hypnotically, intra-articular injection of CDDO-Me should stimulate HMGB1 secretion, worsening CIOA, because in the early stages of CIOA, pyroptosis would be less apparent than in the late OA stages, and distinct cell types involved in this complex pathology would respond differently to Nrf2 activation, and hence to pyroptosis. For example, neutrophils are early players in the destructive process in CIOA and intensively infiltrate the synovium at day 1 of CIOA, while monocytes can migrate to the synovium at day 3 [26] and may contribute more to elevated IL-1β via the HMGB1-mediated inflammasome pathway during the late stage of OA. Inflammasome activation and pyroptosis can be suppressed by increased Nrf2 expression in macrophages [65]. Neutrophils, on the other hand, lack gasdemin-mediated pyroptosis and can nevertheless release IL-1β into tissue compartments to maintain the inflammatory milieu [65,66]. Thus, senescent neutrophils may aggravate not only early, but also late stages of OA, by providing an extra source of IL-1β and, despite being present in low numbers in OA synovial fluid, may enhance the detrimental functions of chondrocytes. Therefore, by decreasing neutrophils senescence with CDDO-Me, we might limit the possibility for elevating IL-1β through the non-canonical and non-lytic pathway in synovium at late OA stage along with an inhibitory effect on HMGB1-meditaed inflammasome activation in macrophages and/or synovial fibroblasts and chondrocytes. As released IL-1β might affect the levels of ROS, lipid ROS, and the lipid peroxidation end product malondialdehyde (MDA) in chondrocytes and synovial fluid [67], and further cartilage degradation and OA progression, the levels of MDA and IL-1β might be important biomarkers to monitor the local effects of Nrf2 activators in clinical trials in the future. In addition, we believe that in such studies, an analysis of the senescence phenotype of neutrophils in blood as an indirect contributor to OA development should also be included. 

As the role of neutrophils in cancer has been recently revisited [68], and Nrf2 has a protective role in cancer prevention and progression [39], it will be very interesting to study the future potential of Nrf2 activators in neutrophil function in cancer. How increased Nrf2 levels may affect total BM niches and granulopoiesis during the progression of joint damage remains to be investigated. Altogether, our data suggest that the Nrf2 activator CDDO-Me can regulate neutrophil senescence during the progression of knee-joint damage.

## 4. Materials and Methods

### 4.1. Chemical Compound

Analytical grade 2-Cyano-3,12-dioxo-oleana-1,9(11)-dien-28-oic acid methyl ester (CDDO-Me; Bardoxolone methyl) was purchased from Sigma-Aldrich (#SMB00376; Sigma-Aldrich, Munich, Germany). The compound has a molecular weight of 505.7 g/moL and chemical structure of the bardoxolone methyl ester, as shown in the PubChem Database (Pub Med CID: 400769; see Supplementary Figure S1). CDDO-Me was dissolved in dimethyl sulfoxide (DMSO; #D8418; Sigma-Aldrich, Munich, Germany) to a final concentration of 1 mg/mL. The stock solution was aliquoted in a volume of 100 µL/tube and stored at −20 °C. 

### 4.2. Animals

Balb/c mice were purchased from Charles River Laboratories (Wilmington, MA, USA) and then bred in the Experimental Animals Facility at the Stephan Angeloff Institute of Microbiology (Sofia, Bulgaria). Mice (female or male, 7-week-old, weight 19–23 g) were kept under standard conditions, and fed with a laboratory diet (29% protein, 13% fat, and 56% carbohydrates) and water ad libitum. All of the experiments were performed under local subcutaneous anesthesia with 1% lidocaine for 15 min. At the end of experiments, the mice were killed by cervical dislocation. The procedures were approved by the National Food Agency (Sofia, Bulgaria) and executed according to the European Union Directive 2010/63/EU for animal experiments (License for Animal Housing No. 352/30.01.2012 (registration No. 11130005); License for Experimental Procedures No. 105/10.07.2014). All of the experiments were conducted in accordance with the ARRIVE criteria (Animal Research: Reporting of In Vivo Experiments) and the principles of the 3Rs (replacement, reduction, and refinement) and under daily control by a certified veterinarian and regular control by the representatives of the National Food Agency. 

### 4.3. Model of Collagenase-Induced Osteoarthritis (CIOA) and Treatment

Osteoarthritis was induced in anesthetized mice, as previously described [26]. Balb/c mice (21.3 ± 2.1 g, 7-week-old; female or male) were injected in the knee cavity with 5 U/10 µL of collagenase from *Clostridium histolyticum* (#C0130, Sigma-Aldrich, Munich, Germany). The enzyme was initially dissolved at a concentration of 1 mg/mL in TESCA buffer (pH 7.4) containing 50 mM TES (N-[Tris(hydroxymethyl)methyl]-2-aminoethanesulfonic acid sodium salt) and 0.36 mM CaCl_2_ at 37 °C in a temperature- and humidity-controlled incubator (Heidolph Incubator 1000, Heidolph Instruments GmbH, Schwabach, Germany). Collagenase was aliquoted, stored at −80 °C, and diluted immediately before each experiment in endotoxin-free phosphate-buffered saline (PBS) containing Ca^2+^ and Mg^2+^ (D-PBS; 0.90 mM CaCl_2_, 2.68 mM KCl, 0.49 mM MgCl_2_·6H_2_O, 136.8 mM NaCl, 15.21 mM Na_2_HPO_4_). The control group of mice were intraarticularly (i.a.) injected with 10 µL of endotoxin-free D-PBS. Under local subcutaneous anesthesia with 1% lidocaine, CDDO-Me was carefully injected in the knee cavity using a Hamilton micro-syringe with a 34-gauge needle (Hamilton, Domat/Ems, Switzerland). The compound was administered twice a week starting at day 7 post-CIOA induction at a dose of 50 µg/knee (*n* = 7 animals/group in at least two experiments). The vehicle control group received 0.01% DMSO/PBS. At day 14, mice were killed and the knee joints were collected and used for histology or for the preparation of synovial tissue extracts. 

### 4.4. Histology

Dissected right knee joints were fixed in 10% paraformaldehyde (PFA)/PBS, and decalcified in 10% ethylenediaminetetraacetic acid (EDTA, #E6758, Sigma-Aldrich, Munich, Germany) for 1 week. They were then dehydrated, embedded in paraffin, cut (4 μm thick; sections harvested at 60 μm interval with total 10 sections/mouse from *n* = 10 mouse/group), and stained using the Safranin-O/Fast-Green technique [69]. Cartilage and joint damage were quantified by the Grade × Stage score system recommended by Osteoarthritis Research International Society International (OARSI) [69]. Each section was evaluated histologically by two independent observers in a blinded manner using a light microscope (Boeco BM-800, Hamburg, Germany) at 20× and 40× magnification and the scores were averaged for the tibia and femur of each mouse/sample. Photos were captured at 40× magnification using a Nikon camera (Nikon Inc, Melville, NY, USA) (see Supplementary Figure S4).

### 4.5. Synovial Tissue Extracts

Synovial tissue extracts were prepared as described previously by Rosengren et al. [70]. The left knee joints (5 mm^2^ size) were collected, placed on ice, and minced to smaller fragments with a scalpel. The fragments were weighed, snap-frozen in liquid nitrogen, and stored at −80 °C until the time of extraction. Ice-cold extraction buffer, consisting of 0.1% Igepal CA-630/PBS (#I8896, Sigma-Aldrich, Munich, Germany) with protease inhibitors (#A32963, Pierce Protease Inhibitor Tablets, Thermo Fisher Scientific, Waltham, MA, USA), was added at a volume of 100 μL per 1 mg of tissue. The samples were prepared on ice using a cordless micro-tube homogenizer (#BAF651000000; SP Bel-Art^®^ Proculture Homogenizer system; Sigma-Aldrich, Munich, Germany) and were disintegrated until only white fibrous insoluble connective or bone tissue remained. After incubation for 20 min on ice, the samples were subjected to centrifugation for 10 min/200× *g* at 4 °C. The supernatant was collected, aliquoted, and stored at −80 °C for downstream analysis. 

### 4.6. Enzyme-Linked Immunosorbent Assay (ELISA)

The level of MMP-9 was measured using the Quantikine ELISA kit (#MMPT90; R&D System, Abingdon, UK) with a detection range of 0.1–5 ng/mL. The level of TNF-α was determined using the using ELISA MAX™ Deluxe kit (#430904; BioLegend, London, UK) with a detection range of 7.8–500 pg/mL. Each sample was collected from one mouse/group and the samples were assayed in duplicate. The concentration (pg/mL) of MMP-9 and TNF-α was calculated from a standard curve of the respective recombinant mouse protein using Gen5 Data Analysis Software (BioTek Instruments, Bad Friedrichshall, Germany).

### 4.7. Cell Isolation and Purification

Bone marrow was collected by flushing the murine tibia and the cell suspension was prepared as previously described [26]. The cells were overlaid on a gradient of 55%, 65%, and 75% Percoll™ (#GE17-5445-01, GE Healthcare Europe, Diegem, Belgium) diluted in 0.15 M NaCl and centrifugated for 40 min/250× g at room temperature. Mature neutrophils, at a 65%/75% interface, were aspirated carefully, washed in PBS, counted on a hemocytometer, and resuspended at 2 × 10^6^ cells/mL in sterile complete Roswell Park Memorial Institute (RPMI) 1640 medium containing 2 mM L-glutamine (#R8758), 10% fetal bovine serum (#F7524), 100 U/ml penicillin, and 100 µg/mL streptomycin (#P4333) (all from Sigma-Aldrich, St. Louis, MO, USA). The cell population consisted of >90% viable cells evaluated by Trypan blue staining, of which 88−90% cells were positive for lymphocyte antigen 6G (Ly6G), assessed using flow cytometry.

Freshly isolated BM cells were subjected to Percoll™ gradient centrifugation followed by CD11b positive selection using MojoSort™ Mouse CD11b Selection Kit (#480109, BioLegend, London, UK). Briefly, 1 × 10^7^ cells/mL were resuspended in 100 µL of PBS containing 0.5% bovine serum albumin (BSA, #A2153, Sigma-Aldrich, St. Louis, MO, USA) and 2 mM EDTA (Sigma-Aldrich, Munich, Germany), and were incubated with 10 µL of biotinylated anti-CD11b antibody (clone M1-70; BioLehend kit) for 15 min at 4 °C. After washing, the cells were incubated with 20 µL of MojoSort™ streptavidin nanobeads for 15 min at 4 °C. Then, the cells were centrifuged at 300× *g*, at 10 °C, washed with 2 mL buffer, resuspended at 1 × 10^8^ cells in 500 µL of buffer, and exposed to the magnet for 5 min at room temperature. CD11b-negative and -positive cells were collected in separate tubes, cell purity was evaluated by flow cytometry, and the suspensions were used for analyses of CXCR4 or β galactosidase expression. 

### 4.8. Analysis of PARP-1 Cleavage

Purified BM neutrophils were seeded at a concentration of 2 × 10^5^ cells/well in a 96-well plate (TPP Techno Plastic Products AG, Trasadingen, Switzerland). The cells were stimulated with 100 ng/mL LPS (#2637, strain O5:B5, Sigma-Aldrich, Munich, Germany) in the presence of increasing concentrations (10, 100, and 1000 nM) of CDDO-Me. Cleaved PARP-1 was determined using the colorimetric Pierce™ Cleaved Parp In-Cell kit (#62219; Thermo Fisher Scientific, Waltham, MA, USA) according to the manufacturer’s protocol described below. After 18 h of incubation at 37 °C, 5% CO_2_, the culture medium was removed and 100 µL of 4% paraformaldehyde (PFA)/PBS (#420801, BioLegend, London, UK) was added to each well and the cells were fixed at room temperature for 15 min. The cells were washed with Tris buffered saline, permeabilized for 15 min at room temperature, and treated with the Quenching Solution containing 1% H_2_O_2_ for 20 min at room temperature. After washing, the plate was incubated with the Blocking buffer for 30 min at room temperature, followed by incubation with primary antibodies against cleaved PARP-1 (1:1000 dilution) and α-Tubulin (1:500 dilution) at 4 °C, overnight. After washing, diluted horseradish peroxidase (HRP) conjugate was added (100 µL/well) for 30 min, at room temperature. Additional cell controls were left without the HRP conjugate to control the non-specific background signal. The specific signal was determined in triplicate for each primary antibody (cleaved PARP-1 and α-Tubulin). The substrate 3,3′,5,5′-tetramethylbenzidine (100 µL/well) was used to visualize the specific interactions. The reaction was stopped after 15 min and the absorbance measured at 450 nm (A450) using an ELISA reader (BioTek Instruments, Bad Friedrichshall, Germany). The fluid was next aspirated and the Green staining was performed to adjust cell number in each well. After incubation with an elution buffer, the absorbance was measured at 630 nm (A630). The results were calculated after initial subtraction of the non-specific background measurements for each condition. The A450 values were normalized to the A630 values from the corresponding well (A450/A630). The average values for cleaved PARP-1 were then normalized to the average values for α-tubulin (cleaved PARP/α-tubulin) and plotted on a graph.

### 4.9. In Vitro Chemotaxis Assay towards SDF-1

Purified BM neutrophils (2 × 10^6^ cells/mL) were pre-treated with CDDO-Me (from 1 nM to 1 µM) for 1 h. The compound was washed off, and the cells were collected, re-counted, and re-suspended in fresh RPMI-1640 medium at a concentration 1.5 × 10^6^ cells/mL. The cells were seeded in trans-well inserts (3 µm pore; Corning™ Sigma-Aldrich, Munich Germany) suitable for 24-well plates. Cell culture medium containing 200 µL of 10 ng/mL recombinant murine SDF-1 (#250-20B; PeproTech, Cambridge, UK) was added to the bottom of the plate. Chemotaxis was allowed to proceed for 6 h at 37 °C/5% CO_2_. The cells were then collected from the plate well and enumerated using BD Trucount™ Tubes (#662415; BD Biosciences, San Jose, CA, USA) containing a known concentration of fluorescent beads. The absolute number of Ly6G+ cells was calculated by dividing the number of cellular events by the number of bead events. The result was then adjusted to the BD Trucount™ bead concentration/tube in order to determine the total neutrophil counts per well.

### 4.10. Flow Cytometry for Phenotyping Cell Surface Expression of Neutrophil Receptors and Necrosis

The cell phenotype and surface receptor expression were determined as previously described [26]. Briefly, BM cells (1 × 10^5^ cells/mL) or freshly purified neutrophils (1 × 10^5^ cells/mL) in 2% BSA/PBS were incubated for 20 min at 4 °C, in the dark, with validated concentrations of fluorochrome-conjugated antibodies against mouse Ly6G (clone 1A8, #127605 or #127653), CD11b (clone M1-70; #101211 or #982606), CXCR4 (clone L276F12; #146507)) and of the corresponding isotype rat control antibodies (all from BioLegend, London, UK). Antibodies were conjugated with the following fluorophores: fluorescein isothiocyanate (FITC), phycoerythrin (PE), PE-Cy7, allophycocyanin (APC), and Alexa Fluor^®^ 647, respectively. After washing, the samples were analyzed by flow cytometry. 

To monitor necrosis, the cells were stained with 1 µL of PI solution (250 µg/mL solution in PBS; 0.25 µg/assay, #ab14083, Abcam, Cambridge, UK) for 3 min, in the dark, at room temperature, and the subjected to analyses. To calculate the fold of LPS-induced necrosis in each sample, we used the following formula:

(1) $$\begin{array}{lll} a & = & \text{\% PI+ cells in the CDDO-treated LPS stimulated group;}\\ b & = & \text{\% PI+ cells in the non-treated LPS stimulated group;}\\ c & = & \text{\% PI+ cells in the CDDO-treated non-stimulated group;}\\ d & = & \text{\% PI+ cells in the non-treated unstimulated group.} \end{array}$$

Fold of LPS-induced necrosis = (a − b)/(c − d)(2)

### 4.11. Flow Cytometry for Intracellular Detection of Nrf2, Phosphorylated p38, and β-Galactosidase

The protocol from our previous study was adapted to detect intracellular proteins [71]. Briefly, purified BM cells (1 × 10^6^ cells/mL) were fixed with 4% PFA/PBS and permeabilized for 15 min at room temperature, in the dark, using a Cytofix/Cytoperm™ kit (#554714, BD Biosciences Pharmingen, San Diego, CA). After washing with 5% BSA/PBS buffer and Fc blocking for 10 min on ice with anti-CD16/32 antibody (#101302; clone 93; 1.0 μg/1 × 10^6^ cells/mL; BioLegend, London, UK), the samples were stained with anti-Nrf2 (1:100 diluted, #12721; clone D1Z9C, Cell Signaling Technology, Leiden, The Netherlands) or anti-β-galactosidase antibody (dilution 1:200, #27198; clone E2U2I, Cell Signaling Technology, Leiden, The Netherlands) or isotype control antibodies (Cell Signaling Technology, Leiden, The Netherlands), followed by staining with a FITC-labeled anti-rabbit IgG (diluted 1:1000, #406403; donkey anti-rabbit IgG with minimal x-reactivity; BioLegend, London, UK) for 1 h, at 4 °C, in the dark, and then they were subjected to flow cytometry analysis. Phosphorylation of p38 was measured as described previously [71] using vthe PE-labelled antibody against pT180/pY182, pp38 (1:100 diluted; clone 4NIT4KK, #612565, eBiosciences, Hatfield, UK), purified antibody against total protein p38 (1:100 diluted; clone Poly6224; #622403, BioLegend, London, UK) and FITC-labelled anti-rabbit antibody (1:500 diluted; #406403; donkey anti-rabbit IgG with minimal x-reactivity; BioLegend, London, UK). The mean value of the phosphorylated p38 (pp38) protein was normalized to the mean value of total p38 protein, as described in our previous study [71].

### 4.12. Flow Cytometry Data Analysis

After the acquisition of at least 20,000 cell counts/sample and live/dead cell discrimination at BSR II flow cytometer, the data were obtained using the BD FACSDiva v6.1.2 Software (Becton Dickinson GmbH, San Jose, CA, USA). The data were also analyzed by Flowing Software 2.2 (Cell Imaging Core, Turku Centre for Biotechnology, Turku, Finland) or Cyflogic software 1.2.1 (CyFlo Ltd., Turku, Finland).

### 4.13. Immunofluorescence for Detection of β-Galactosidase and CXCR4

BM neutrophils were isolated, purified by gradient centrifugation, and subjected to CD11b cell enrichment by magnet sorting. Ly6G+CD11b+ neutrophils (2 × 10^6^ cells in 100 µL) were cultured in 8-well glass slide chambers (Ibidi, Martinsried, Germany) in the absence or presence of 1 µM CDDO-Me for 6 h at 37 °C, 5% CO_2_. The cells were washed, stained for 15 min with PerCP-Cy5-5 labelled antibody against Ly6G (clone 1A8, #127653, BioLegend, London, UK), and washed and fixed with 4% PFA/PBS for 10 min at room temperature. The unspecific binding was blocked with 0.3% Triton X-100 (#T9284, Sigma-Aldrich, Munich, Germany) for 1 h at room temperature, followed by incubation with the APC labelled antibody against CXCR4 (#146507; clone L276F12; BioLegend, London, UK) and purified rabbit antibody against β-galactosidase (1:250 diluted in 1% BSA/0.3% Triton-X 100/PBS; #27198; clone E2U2I, Cell Signaling Technology, Leiden, The Netherlands) overnight at 4 °C. After washing, the cells were incubated with FITC-labelled anti-rabbit antibody (1:1000 diluted in 1% BSA/0.3% Triton-X 100/PBS; #406403; donkey anti-rabbit IgG with minimal x-reactivity; BioLegend, London, UK) for 1 h at room temperature, in the dark. After washing, the 8-well chambers were removed and a Vectashield Antifade Mounting Medium with 4′,6-diamidino-2-phenylindole (DAPI) (#H-1200; Vector Laboratories, USA) was drop added to the glass slide, then covered with a coverslip, and left for 10 min in the dark. The slide was subjected to fluorescent microscopy using a Leica DM6 microscope with a DFC7000 T color microscope camera and the LAS X 5.0.2 software at a magnification of 60× (Leica Microsystems; RSR Ltd., Sofia, Bulgaria). The photos were taken from five different fields of each sample using appropriate filters and exposure, and were then merged. Photo’s analysis was performed using Image J software (National Institutes of Health, University of Wisconsin) after defining the threshold of each signal—red, blue, and green. The staining intensity of each signal was presented in pixels and compared between groups. Cell counts were obtained using Image J plug-in for counting. 

### 4.14. Determination of β-Galactosidase Activity

BM cells were isolated from the femur and tibia of Balb/c mice and purified by Percoll gradient centrifugation [26]. Beta-galactosidase activity was determined according to the protocol of Debacq-Chainiaux et al. [72]. Briefly, the cells were seeded at 24-well plates at a concentration of 2 × 10^6^ cells/mL and were cultured in the presence of 0.01% DMSO (Vehicle) or 1 µM CDDO-Me. After 6 h, the cells were fixed with 0.2% Glutaraldehyde/dH_2_O (#354400; Sigma-Aldrich, Munich, Germany) for 10 min, washed twice with PBS, and incubated with the chromogenic buffer containing 150 mM NaCl, 2 mM MgCl_2_·6H_2_O; 1 mg/mL X-Gal (#203782, 5-Bromo-4-chloro-3-indolyl-β-D-galactopyranoside, Calbiochem, Munich, Germany), and 0.4 mM Nitrotetrazolium Blue Chloride (NBT) (#11585029001, Roche, Munich, Germany) dissolved in phosphate buffer (pH 7.4; 500 mM K_2_HPO_4_·3H_2_O; 500 mM KH_2_PO_4_) for 18 h. The fixed cells were rinsed with 80% methanol/dH_2_O and air dried. The cells positive for beta-galactosidase were stained in blue. Each well was examined using a Leica DMi8 inverted microscope at 10× and 20× magnifications using a Flexacam C1 camera (Leica Microsystems; RSR Ltd., Sofia, Bulgaria). The photos were captured and the cells showing β-galactosidase activity (blue staining) were detected and counted using Image J software. 

### 4.15. Statistical Analyses

Statistical analysis was performed using InStat 3.0 and GraphicPad Prism 5.0 (GraphPad Software, La Jolla, CA, USA). The data were represented as mean ± standard error (SEM) or mean ± standard deviation (SD). ANOVA tests were used for data analysis of non-parametric or a low number of samples. For other data, the differences in the mean values between groups were analyzed using the two-tailed Student’s t-test. Every possible comparison between all of the studied groups was evaluated and then presented on the graphs. The differences were considered significant when *p* < 0.05. Pearson’s coefficient was used for the correlation analysis. The table for degree of freedom was generated using the Real-Statistic plug in.

## Data Availability

Not applicable.

## Supplementary Materials

The following supporting information can be downloaded at: https://www.mdpi.com/article/10.3390/ijms24108775/s1. Reference [73] is cited in the supplementary file.
