# Supplementary material for: The Triterpenoid Nrf2 Activator, CDDO-Me, Decreases Neutrophil Senescence in a Murine Model of Joint Damage
Source: Int J Mol Sci. 2023 May 15;24(10):8775. doi: 10.3390/ijms24108775 (PMC10217923; doi:10.3390/ijms24108775)
Supplement: Supplementary file 1 [file ijms-24-08775-s001.zip › ijms-2334948-supplementary.pdf]

International Journal of
Molecular Sciences

MDPI

The Triterpenoid Nrf2 Activator, CDDO-Me, Decreases Neutrophil Senescence in a Murine Model of Joint Damage

Kristiana M. Amirova [1,2,†], Petya A. Dimitrova [3,†], Milena N. Leseva [3], Ivanka K. Koycheva [1,2], Albena T. Dinkova-Kostova [4,5] and Milen I. Georgiev [1,2,*]

[1] Laboratory of Metabolomics, The Stephan Angeloff Institute of Microbiology, Bulgarian Academy of Sciences, 4000 Plovdiv, Bulgaria; kristiana.amirova@gmail.com (K.M.A.); vkoy4eva@abv.bg (I.K.K.)
[2] Center of Plant Systems Biology and Biotechnology, 4000 Plovdiv, Bulgaria
[3] Department of Immunology, The Stephan Angeloff Institute of Microbiology, Bulgarian Academy of Sciences, 1113 Sofia, Bulgaria; petya_dimitrova@web.de (P.A.D.); mlesseva@gmail.com (M.N.L.)
[4] Division of Cellular and Systems Medicine, School of Medicine, University of Dundee, Dundee DD1 9SY, UK; a.dinkovakostova@dundee.ac.uk (A.T.D.-K.)
[5] Department of Medicine and Pharmacology and Molecular Sciences, School of Medicine, Johns Hopkins University, Baltimore, MD 21205, USA
* Correspondence: milengeorgiev@gbg.bg; Tel: +359-32-64-24-30
† These authors contributed equally to this work.

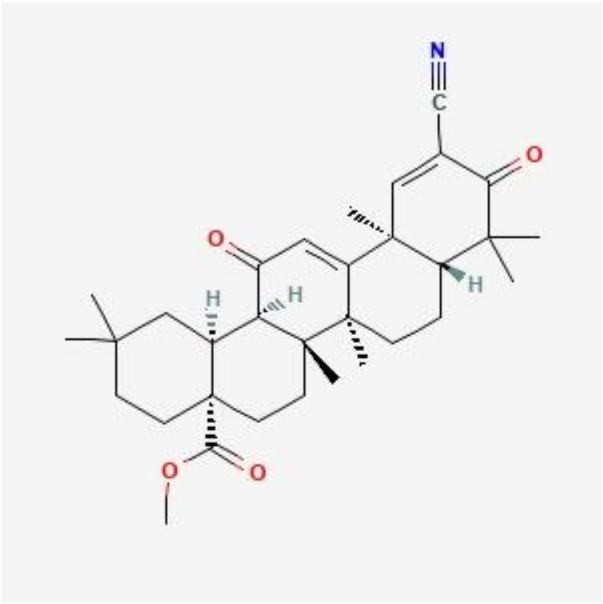

Figure S1. Chemical 2D structure of CDDO-Me according to the PubChem Database. The compound has the PubChem CID - 400769.

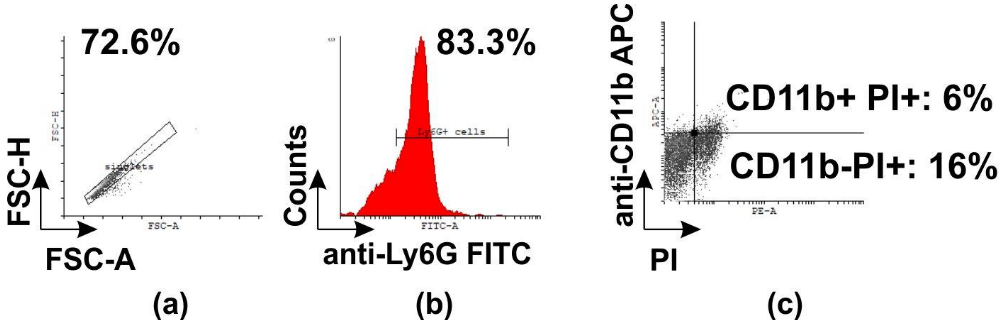

Figure S2. Gating strategy to determine CD11b+PI+ and CD11b-PI+ cells. BM cell were collected and cultured as described in Material and Method section for 24 hours, then washed and used for flowcytometry analyses (**a**) Dot-plot showing the gate of singlets. (**b**) Histogram demonstrating Ly6G+ cell gate. (**c**) Dot-plot with the quadrant gate was used to determine the frequency of CD11b+PI+ cells and CD11b-PI+ cells.

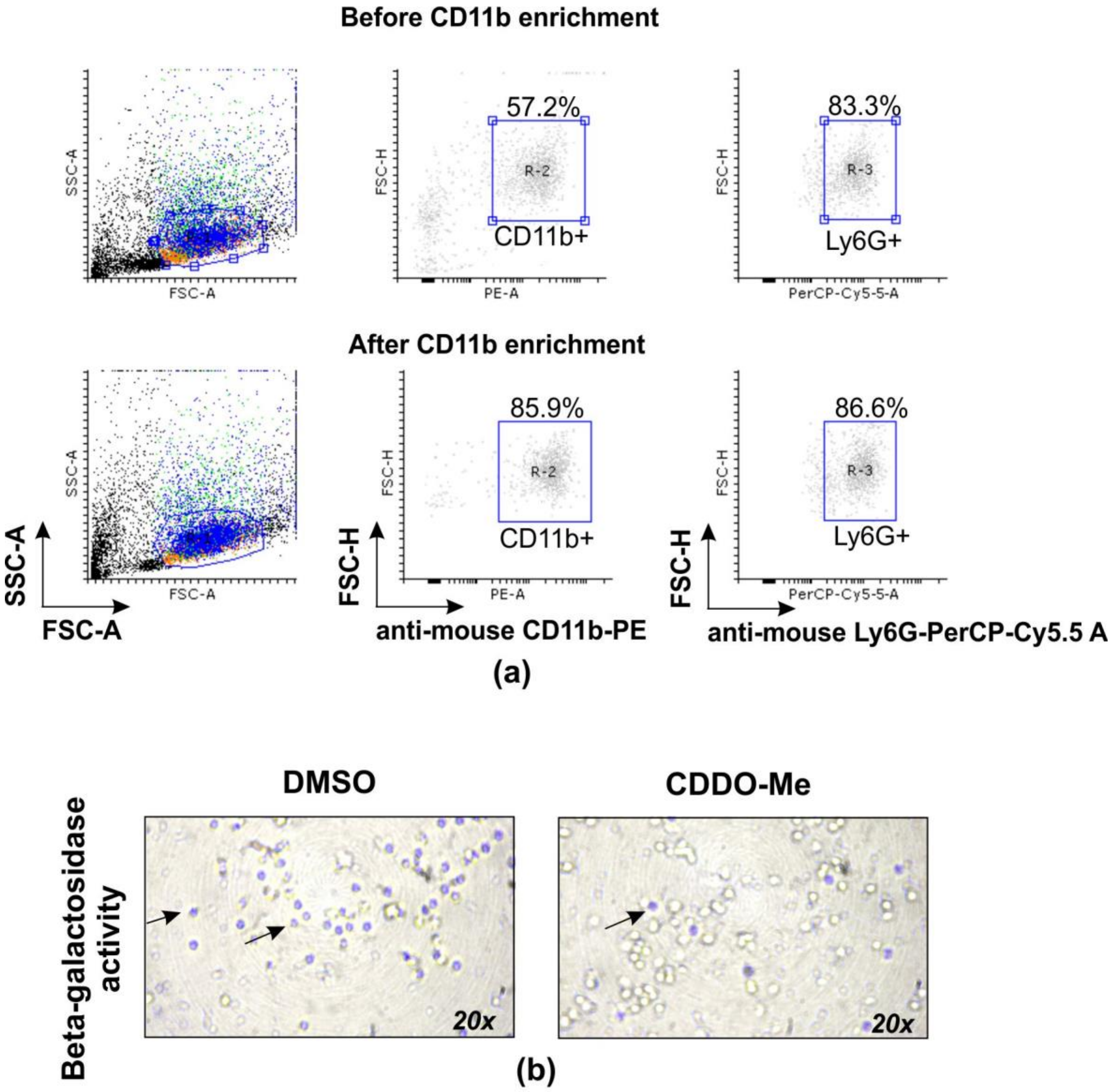

Figure S3. Enriched CD11b+ cells from murine BM Ly6G+ cells. **(a)** BM cells were purified using density centrifugation [42] and then were subjected to magnet enrichment using CD11b+ isolation kit (MojoSort™ Mouse CD11b Selection Kit (#480109, BioLegend, London, UK). The cells were then subjected to flow cytometry analyses using antibodies against CD11b PE labelled and/or Ly6G PerCP-Cy5.5 labelled. **(b)** Photomicrographs showing staining of cells for β-galactosidase activity. The method was performed on 96-well plate and each well was examined by Leica DMi8 inverted microscope at *10*× and *20*× magnifications using Flexacam C1 camera (Leica Microsystems; RSR Ltd, Sofia, Bulgaria). The photos were captured and the cells showing β-galactosidase activity (blue staining) were detected and counted by Image J software.

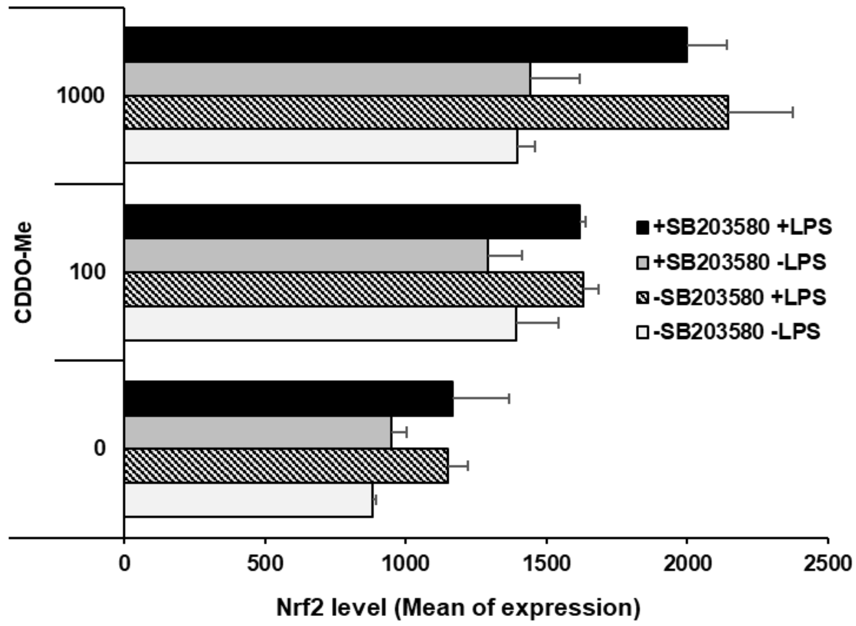

Figure S4. Effect of p38 MAPK kinase inhibitor SB203580 on cytoplasmic Nrf2 level. Bone-marrow derived neutrophils were untreated/treated with CDDO-Me at concentrations 100 and 1000 nM in the presence of 100 ng/mL LPS and in the presence/absence of 100 nM p38 MAPK inhibitor SB203580 (#ab120162; Abcam, Cambridge, UK) for 6 h. The concentration of the inhibitor was selected according to the publication of Xu et al. (2013) [73]. The level of Nrf2 was measured by flow cytometry as described in Material and Methods section 4.11. The data were obtained using BSR II flow cytometer with BD FACSDiva v6.1.2 Software (Becton Dickinson GmbH, San Jose, CA, USA) and analyzed by Cyflogic software 1.2.1 (CyFlo Ltd, Turku, Finland). The data represent mean±SEM of one experiment including 3 mice; samples run in triplicate (n=3/group).

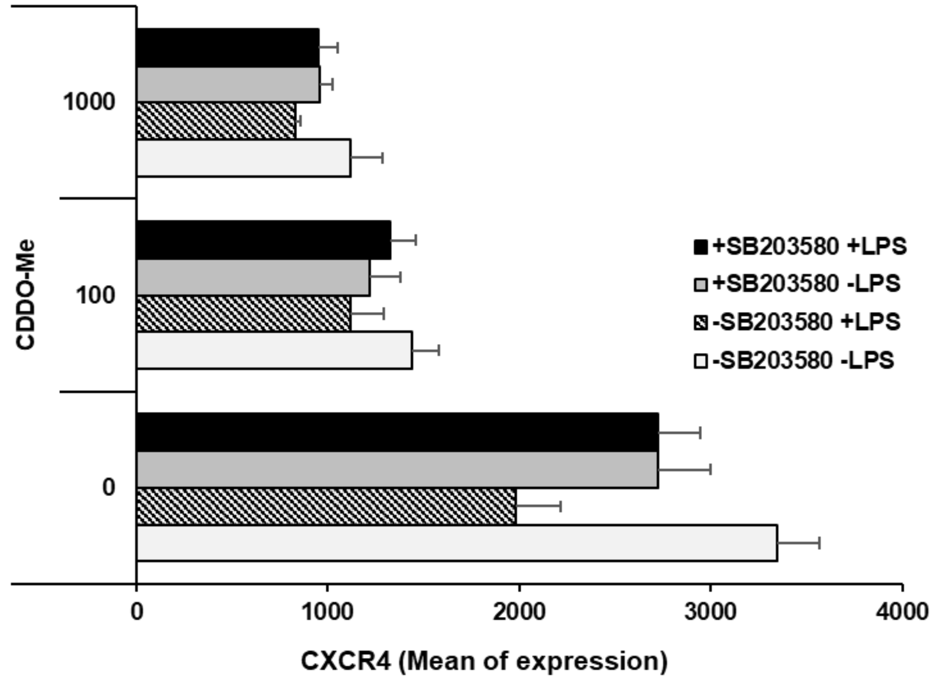

Figure S5. Effect of p38 MAPK kinase inhibitor SB203580 on CXCR4 expression. Bone-marrow derived neutrophils were untreated/treated with CDDO-Me at concentrations 100 and 1000 nM in the presence of 100 ng/mL LPS and in the presence/absence of 100 nM p38 MAPK inhibitor SB203580 (#ab120162; Abcam, Cambridge, UK) for 6 h. The concentration of the inhibitor was selected according to the publication of Xu et al. (2013) [73]. The expression of CXCR4 was measured by flow cytometry as described in Material and Methods section 4.10. The data were obtained using BSR II flow cytometer with BD FACSDiva v6.1.2 Software (Becton Dickinson GmbH, San Jose, CA, USA) and analyzed by Cyflogic software 1.2.1 (CyFlo Ltd, Turku, Finland). The data represent mean±SEM of one experiment including 3 mice; samples run in triplicate (n=3/group).
